# Removal of hexavalent chromium by facultative anaerobic strains *Bacillus* sp. S9 and *Enterobacter* sp. Z11 from a mining site microbial mat

**DOI:** 10.1007/s13205-026-04841-9

**Published:** 2026-05-15

**Authors:** Mohammad Tariq Ali Khan, Wael Ismail, El-Said I. El-Shafey, Raeid M. M. Abed

**Affiliations:** 1 Biology Department, College of Science, Sultan Qaboos University, P. O. Box: 36, Al Khoud, PC 123 Sultanate of Oman; 2 Center of Environmental and Biological Studies, Arabian Gulf University, Manama, Bahrain; 3 Chemistry Department, College of Science, Sultan Qaboos University, P. O. Box: 36, Al Khoud, PC 123 Sultanate of Oman

**Keywords:** Hexavalent chromium, Microbial mat, Facultative anaerobes, Biosorption, Bioreduction, Bioaccumulation

## Abstract

Hexavalent chromium [Cr(VI)] removal was evaluated using two facultative anaerobic bacteria isolated from a microbial mat near a chromite mining site: *Bacillus* sp. S9 (Gram-positive) and *Enterobacter* sp. Z11 (Gram-negative). Both strains effectively removed Cr(VI) under anaerobic conditions, with total removal efficiencies of 98.3 ± 0.6% and 97.2 ± 0.9%, respectively. The bacteria reduced Cr(VI) to trivalent chromium [Cr(III)], with this reduction occurring extracellularly in the supernatant, on cell surfaces, and intracellularly. Chromate reductase assays and spectroscopic analyses confirmed the predominant biological reduction of Cr(VI) to Cr(III), with minor formation of elemental chromium [Cr(0)]. Surface analyses showed Cr deposition on the surface of *Bacillus* sp. S9 (13.3 ± 0.5%) and *Enterobacter* sp. Z11 (33.9 ± 2%), with functional groups such as OH-NH, methyl-C-H, C = O/COO, C-N, N-H, PO_4_^3-^, metal–O, and Cr(VI)–O bonds contributing to biosorption. Both strains secreted approximately two-fold more extracellular polymeric substances (EPS) after exposure to Cr(VI), thereby enhancing metal binding. Intracellular Cr accumulation was also observed, with a total amount of 3.9 ± 0.3 mg L⁻¹ in *Bacillus* sp. S9 and 6.4 ± 0.3 mg L⁻¹ in *Enterobacter* sp. Z11. These results demonstrate that facultative anaerobes from microbial mats effectively reduce, adsorb, and accumulate Cr species, underscoring their potential application in bioremediation of Cr-contaminated environments under varying oxygen conditions.

**Supplementary Information:**

The online version contains supplementary material available at 10.1007/s13205-026-04841-9.

## Introduction

Cr(VI) contamination can result from different industrial wastes, mining activities, use of Cr-based fertilizers, and weathering of ultramafic Cr-rich rocks (Al-Battashi et al. 2016; Banerjee and Dey 2024; Dey 2022). Due to its high solubility, Cr(VI) can readily migrate through soil and water, leading to persistent groundwater contamination and associated risks to human health and ecosystems (Tchounwou et al. 2012). Bioremediation through bacterial reduction of Cr(VI) to less toxic and less mobile Cr(III) is a cost-effective and environmentally friendly sustainable approach (Aishwarya et al. 2024; Deo et al. 2024; Dey 2022; Pushkar et al. 2021). This process can occur under aerobic and anaerobic conditions, involving diverse microorganisms and biochemical pathways (Durán et al. 2018; Huang et al. 2023; Martins et al. 2010; Aishwarya et al. 2024; Deo et al. 2024). In anaerobic environments such as subsurface soils, sediments, aquifers, and landfills, anaerobic microorganisms including sulfate-reducing bacteria, iron-reducing bacteria, and facultative anaerobes can contribute to dissimilatory Cr(VI) reduction (Tchounwou et al. 2012; Zha et al. 2024). Reduction may occur either enzymatically, where Cr(VI) serves as a terminal electron acceptor, or indirectly via reactions with reduced metabolites such as H_2_S and Fe(II) (Huang et al. 2023; Li et al. 2023; Wang et al. 1989). Facultative anaerobic bacteria are particularly promising for Cr(VI) bioremediation due to their ability to function under fluctuating oxygen conditions, providing greater operational flexibility in environmental applications (Cheung and Gu 2007; Wang and Shen 1995; Zha et al. 2024). These bacteria can switch electron acceptors, such as oxygen and Cr(VI), enabling enzymatic reduction of Cr(VI) to Cr(III), depending on environmental conditions (Baldiris et al. 2018; Thatoi et al. 2014). In addition, functional groups on their cell surfaces facilitate biosorption and immobilization of Cr(VI), which often precedes enzymatic reduction (Zha et al. 2024). Facultative anaerobes simplify Cr(VI) bioremediation by eliminating the need for strict anaerobic conditions, reducing system complexity and cost. Consequently, they are increasingly explored for bioremediation applications in anaerobic environments.

Numerous facultative anaerobic bacteria capable of reducing Cr(VI) under anaerobic conditions have been reported (Banerjee and Dey 2024; Dey 2022; Narayani and Shetty 2013). For example, *Pseudomonas* sp. and *Aeromonas* sp. were among the first strains shown to remove Cr(VI) anaerobically (Romanenko and Koren’Kov 1977; Wang et al. 1989). Other facultative anaerobes such as *Enterobacter cloacae* and *E. coli* could reduce 100 mg L^− 1^ Cr(VI) involving chromate reductases (Wang et al. 1989; Mohamed et al. 2020). *Bacillus* spp. (e.g., *B. subtilis* and *B. aureus*), and *Pseudomonas* spp. (e.g., *P. aeruginosa* and *P. putida*) are also known to tolerate high concentrations of Cr(VI) and achieve substantial removal under anaerobic conditions (Narayani and Shetty 2013; Padma et al. 2023; Upadhyay et al. 2017). Additional Cr(VI)-removing facultative anaerobes have been reported from genera such as *Shewanella*, *Klebsiella*, and *Staphylococcus* (Banerjee and Dey 2024; Dey 2022; Narayani and Shetty 2013). Although previous studies have characterized removal rates and identified enzymatic reduction and biosorption as key mechanisms in facultative anaerobic Cr(VI) bioremediation, relatively few have examined these processes in bacteria from unique ecological niches such as microbial mats. Microbial mats are stratified with steep oxygen gradient, and are a rich source of facultative anaerobes (Bender 2004; Phillips and Bender 1995). Recently, microbial mats from Cr mining areas in Oman have been shown to host mixed bacterial communities capable of removing Cr(VI) under aerobic conditions (Abed et al. 2020; Khan et al. 2025a). Efficient Cr(VI)-removing isolates affiliated with *Enterobacter*, *Bacillus*, and *Cupriavidus* were obtained, exhibiting tolerance to 2000 mg L^− 1^ Cr(VI) and employing biosorption and bioreduction mechanisms to remove Cr(VI) under aerobic conditions (Abed et al. 2020; Khan et al. 2025a). Subsequent studies demonstrated that the same mat communities could remove Cr(VI) under anaerobic conditions, with enrichment of obligate and facultative anaerobic bacteria (Khan et al. 2025b). However, the mechanisms underlying anaerobic Cr(VI) reduction by these mat-derived bacteria, the relative contributions of different removal processes (i.e. biosorption, bioreduction and bioaccumulation), and the distribution of Cr species across different compartments (supernatant, cell surface, and intracellular fractions) remain poorly understood.

To address these gaps, this study conducted an in-depth investigation of two facultative anaerobic bacterial strains isolated previously from an aquatic microbial mat found adjacent to a chromite mining excavation pit in Oman (Khan et al. 2025a) for their ability to grow and remove Cr(VI) under anaerobic conditions. Employing an integrated biochemical and analytical approach, we quantified the contribution of bioreduction: enzymatic reduction of toxic Cr(VI) to less toxic Cr(III), which may occur on the cell surface or within the cells; biosorption: a metabolism-independent process where Cr(VI) ions bind passively to functional groups on the bacterial cell surface or EPS, and intracellular bioaccumulation: a metabolism-dependent process involving active transport of Cr(VI) into living cells. We clarify the Cr(VI) removal mechanisms and performance of mat-derived isolates, advancing understanding of Cr(VI) bioremediation in subsurface environments.

## Materials and methods

### Sampling of microbial mats and isolation of facultative anaerobes

The original microbial mats were collected from a Cr(VI)-contaminated aquatic body in an excavation pit found near a chromite mining site in Nakhal, Oman (N 23°24.0079’ E 57°44.5321’, Abed et al. 2020). The air temperature in the sampling site ranged between an average of 25 °C in winter, but could reach 45 °C in hot summers. The salinity of the site water was 2%, pH = 9.05 and temperature = 25 ± 0.1 °C at the time of sampling. To isolate Cr(VI)-removing bacteria, a slurry enrichment was performed by adding 0.5 g of the microbial mat (the layer at 2–3 mm depth) to 30 mL of sterile site water in presence of 100 mg L^− 1^ of Cr(VI) in 160 mL culture bottles. All enrichments were set up in replicates and incubated for 30 days at 30 °C under aerobic conditions. Bacterial growth was monitored visually and by measuring optical density at 600 nm (OD_600_) every 5 days. Following visible growth, 100 µL of each enrichment culture was streaked onto Luria–Bertani (LB) agar plates supplemented with 1 mg L^− 1^ of Cr(VI) to isolate single colonies. This plating procedure was repeated thrice to ensure reproducibility and ability of the isolates to grow in the presence of Cr(VI). Pure colonies were obtained after multiple transfers onto LB agar plates supplemented with 1 mg L^− 1^ of Cr(VI). Sixteen bacterial strains were obtained and identified by 16 S rRNA gene sequencing, and were tested for growth across Cr(VI) concentrations of 1–2000 mg L^− 1^ (Khan et al. 2025a). The isolated strains were then tested for their ability to grow under anaerobic conditions in LB medium amended with 1 mg L^− 1^ of Cr(VI). Anaerobic conditions were established by flushing the sealed culture bottles with nitrogen gas for 15 min. All growth experiments were performed in triplicate, and growth was assessed by OD_600_ measurements after 48 h of incubation at 30 °C. Two facultative anaerobes, identified using 16S rRNA sequencing as *Bacillus* sp. S9, and *Enterobacter* sp. Z11, showed significant growth under anaerobic conditions compared to uninoculated controls (*p* < 0.01, one-way ANOVA followed by Tukey’s post-hoc test), as well as robust growth under aerobic conditions. These results confirmed their facultative anaerobic nature and their potential for Cr(VI) removal under varying oxygen levels. The sequences of the two selected strains were previously deposited in the GenBank under the accession numbers PX136962, and PX136967 for *Bacillus* sp. S9, and *Enterobacter* sp. Z11, respectively (Khan et al. 2025a).

### Inoculum preparation and maintenance of anaerobic conditions

For all subsequent experiments, inoculum was prepared by growing the strains in 10 mL LB medium amended with 1 mg L^− 1^ Cr(VI) in 15 mL culture tubes at 35 °C under anaerobic conditions. Growth was measured using a spectrophotometer (DR 3900, HACH, Germany) at 600 nm each day for three days until the OD values reached 1.50 and 1.65 for *Bacillus* sp. S9 and *Enterobacter* sp. Z11, respectively. The biomass dry weight reached 86 g L^− 1^ for *Bacillus* sp. S9 and 91 g L^− 1^ for *Enterobacter* sp. Z11, with dry weight determined after drying the samples at 60 °C for 24 h to ensure consistent and accurate standardization. For further experiments, the inoculum size was standardized across replicates to achieve comparable initial cell densities.

The anaerobic conditions in all further experiments were created by purging the tightly sealed bottles/tubes with nitrogen gas for 15 min. To validate the maintenance of anaerobic conditions throughout the experiments, methylene blue was used as a redox indicator and was added at the concentration of 0.1 mg L^− 1^ to separate control bottles/tubes at the beginning and at the end of the experiment. Methylene blue remained blue under aerobic conditions, but turned colorless in the absence of oxygen, indicating anaerobic conditions (Supplementary Fig. S1). Methylene blue was not added to any experimental bottle/tube due to its negative effect on bacterial cells. Additionally, dissolved oxygen (DO) was also measured (APERA DO 850 instrument, China) in the medium prepared under the same experimental conditions to confirm anaerobic conditions and was found to be ≤ 0.2 mg L^− 1^ at the beginning of the incubation and near-zero levels on following days.

### Growth characteristics of the isolates

Growth of the bacterial strains was tested at various temperatures (25, 35, and 45 °C), pH values (3, 5, 7, 9, and 11), and salinities (1.5%, 3%, and 5%) to optimize growth conditions. The range of parameters was selected to mimic the actual conditions of the sampling site, accounting for seasonal changes and variations in water level. Biotic (only bacteria, no Cr(VI)), and abiotic (only Cr(VI), no bacteria) control cultures were run in parallel under the same conditions. All experiments were conducted in 15 mL culture tubes, each containing 1% inoculum (i.e., 50 µL containing ca. 4.5 ± 0.1 mg biomass), 5 mL of LB medium, and 1 mg L^− 1^ Cr(VI). Anaerobic conditions were maintained as described above. All tubes were incubated at 35 °C, unless otherwise specified, for 4 days at 120 rpm. All experiments were run in triplicate.

To compare the growth, and maximum tolerance of Cr(VI) by both strains under aerobic and anaerobic conditions, they were grown at different Cr(VI) concentrations (1, 10, 20, 50, and 100 mg L^− 1^) for 4 days in 30 mL of LB medium. Based on the optimum growth conditions for both strains, the pH was maintained at 9 and the temperature at 35 °C under anaerobic conditions, whereas pH was maintained at 7 and temperature at 30 °C under aerobic conditions (Khan et al. 2025a). The inoculum size in all experiments was 1% (i.e., 300 µL containing ca. 27.0 ± 0.6 mg of bacterial cells). Growth was measured spectrophotometrically at 600 nm each day using a spectrophotometer (DR 3900, HACH, Germany). All incubations were conducted in triplicate.

The logistic growth model (Zwietering et al. 1990) was applied on the data obtained from the above experiment to characterize the growth dynamics of *Bacillu*s sp. S9 and *Enterobacter* sp. Z11. The following equation was applied:

$$\:\mathrm{y(t)}=\frac{A}{1+\mathrm{e}\mathrm{x}\mathrm{p}(\frac{4\mu\:max}{A}\left(\lambda\:-t\right)+2)}$$

where *y(t)* is the growth (OD_600_) at time t, A is the maximum population size, µmax is the max growth rate, *λ* is the lag time and t is time. The logistic model was fit to the growth curves using nonlinear regression techniques. Model fits provided quantitative insights into how Cr(VI) concentrations and oxygen availability influenced microbial growth kinetics, revealing dose-dependent suppression of growth rates and capacities, delayed growth phases, and differences between aerobic and anaerobic lifestyles.

### Cr(VI) removal experiment

For mechanistic experiments, minimal medium was used due to its defined and controlled nutrient composition, which minimizes interference from complex organic compounds present in rich media such as LB medium. This allows for clearer interpretation of microbial Cr(VI) bioreduction processes, and better mimics natural environmental conditions. Both isolates were grown in 50 mL of minimal medium with glucose (2 g L^− 1^) as a carbon source and tryptophane as a nitrogen source (2 g L^− 1^) at pH 9 in the presence of 50 mg L^− 1^ Cr(VI) as described earlier (Rahman and Thomas 2021). The medium was inoculated with 1% inoculum (i.e., 500 µL containing ca. 45.0 ± 1 mg biomass) of each strain. The minimal medium was prepared by adding 6 g Na_2_HPO_4_, 3 g KH_2_PO_4_, 3 g NaCl and 1 g NH_4_Cl in 1000 ml distilled water. The medium was autoclaved, and then 1 ml of 1 M MgSO_4_ stock and 0.1 ml of 1 M CaCl_2_ stock were added. The concentration of 50 mg L^− 1^ was selected to allow reliable detectability of Cr removal by biosorption, bioreduction, and bioaccumulation. All experiments were conducted in triplicate in 160 mL culture bottles at 35 °C with shaking at 120 rpm for 7 days. The anaerobic conditions in the tightly sealed bottles were maintained as described above. Abiotic controls were included and incubated under the same conditions.

At the end of the experiment, the cell biomass was separated by centrifugation at 5000 rpm for 5 min (Eppendorf, 5804R, Germany) and washed twice with distilled water to obtain the unbound Cr on the surface. The washed cells were then incubated in 5 mL of 0.1 M HCl to release the surface-adsorbed Cr (desorption). To determine the intracellular Cr, the cells were subjected to digestion in 1% HNO_3_. The concentrations of Cr(VI) and Cr(III) were then detected in the supernatant, on the cell surface, and inside the cells. The concentration of Cr(VI) was measured using a ready-made kit provided by TRACE–HT22 chromium hexavalent lot code AA7A0727 (Trace2O, UK) at 540 nm with a spectrophotometer (DR 3900, HACH, Germany). The total Cr concentration in the supernatant was determined using inductively coupled plasma-optical emission spectroscopy (ICP-OES) (Optima 8000DV, Perkin Elmer, USA). The amount of freshly generated Cr(III) was calculated by subtracting the concentration of Cr(VI) from the total Cr (Khan et al. 2025a).

### X-ray photoelectron spectroscopy (XPS) analysis and enzyme assay

To confirm the reduction of Cr(VI) to Cr(III) by the cell biomass, XPS analysis and chromate reductase enzyme assay were performed. XPS was used to determine the chemical state of Cr on the cell surface. The collected cell pellet was dried at 50 °C for 24 h, and the dried biomass (10 mg) was submitted to the Structural Lab facility at Sultan Qaboos University for XPS analysis. Surface characterization was performed using a multi-probe XPS machine (Omicron Nanotechnology, Germany), operated at 15 kV with an Al Kα (hv = 1486.6 eV) radiation source. The XPS results were analyzed using CasaXPS software version 2.3.26 (Casa Software Ltd, UK), applying a Shirley-type background for the analysis. The binding energy of the spectra was calibrated using the C 1 s peak at 284.6 eV.

For the chromate reductase enzyme activity, the cells grown in the presence of 50 mg L^− 1^ Cr(VI) were disrupted by ultrasonication (Bandelin, DT 510 H, Germany) as described before (Camargo et al. 2003). The sonicated samples were then centrifuged and filtered (0.22 μm) to produce cell-free extracts (CFE). The protein content in the CFE was estimated using Bradford assay (Bradford 1976). The contact time was optimized before the actual reaction, and it was found to be 30 min. The reaction mixture for the enzyme assay contained 1 mg L^− 1^ Cr(VI) in 0.8 mL of 100 mM phosphate buffer, pH 7.0. After 5 min of pre-incubation at 35 °C, the reaction was initiated by adding different volumes of the CFE (0.5–2.0.5.0 mL), containing between 0.04 and 0.33 mg protein, depending on the used volume and the bacterial strain. Cr(VI) reduction was measured at the end of the incubation using the above-mentioned kit (Trace2O, UK) at 540 nm after 30 min. CFE-free incubations were maintained as controls. The specific activity of an enzyme, defined as the amount of Cr(VI) reduced per unit time per milligram of total protein present in the CFE (µmol/min/mg or U/mg) was calculated (Camargo et al. 2003). This measurement served as an important measure of the catalytic activity of the enzyme relative to the protein content in the preparation. Higher specific activity indicates a purer and more active enzyme sample.

### Determination of surface-adsorbed Cr

To investigate the adsorption of Cr on the surface of the bacteria, Scanning Electron Microscopy with Energy-Dispersive X-ray spectroscopy (SEM-EDX), and elemental mapping were performed on cell biomass. Additionally, Fourier-transform infrared spectroscopy (FTIR) was used to find out the functional groups involved in the biosorption process. The production of EPS, which are known to play a vital role in biosorption, was assessed. For SEM-EDX, Karnovosky fixative was added to the harvested bacterial cells, and kept at 4 °C for 4 h. The fixation buffer was removed by centrifuging at 5000 rpm for 5 min and sodium cacodylate buffer was added to the cells and kept at 4 °C overnight. The buffer was removed and washed using 100%, 90%, 70%, and 50% ethanol. The cells were dried at room temperature and fixed on aluminum stubs, platinum coated in vacuum and analyzed using SEM. Elemental analysis was performed for carbon, oxygen, and Cr using mapping by SEM–EDX (EDX: Jeol JSM-7600, USA).

For FTIR analysis, bacterial cells were dried at 50 °C for 24 h. Dried biomass was analyzed using a FT-IR ALPHA II Platinum ATR spectrometer (Bruker, Germany) to identify functional group changes related to Cr biosorption. Spectra normalization was done by baseline correction setting the C–H stretching peak at 2923 cm⁻¹ as reference, assuming no change after Cr(VI) treatment. This normalization was applied consistently to all samples for accurate comparison. To evaluate EPS involvement, EPS was extracted from cultures incubated with or without 50 mg L⁻¹ Cr(VI) anaerobically for 7 days (Bales et al. 2013). The cell pellet was centrifuged, and EPS was isolated, dried at 22 °C for 2 h, and weighed. Protein and carbohydrate contents of EPS were measured (Bradford 1976; DuBois et al. 1956).

### Cr(VI) bioaccumulation

To determine the intracellular accumulation of Cr, Transmission Electron Microscopy (TEM) (JEOL, TEM 1200EX, Japan) and Scanning Transmission Electron Microscopy with Energy-Dispersive X-ray spectroscopy (STEM-EDX) (JEOL, JEM 2100 F, Japan) were employed. The sample preparation for TEM and STEM followed previously described protocols (Zakaria et al. 2007). The cells were washed with phosphate-buffered saline (pH 6.8). The cell pellet was fixed using HistoGel (Epredia, HG-4000-012, Fischer Scientific, U.K.) for 24 h at 4 °C, after 24 h the cells were fixed with osmium tetroxide (2%) and potassium ferrocyanide (1%) in 0.1 M carbonate buffer for 1 h, and then washed with 0.1 M carbonate buffer and 0.1 M Na_2_-acetate buffer (pH 5.2) before bloc staining for 1 h. With the help of ultramicrotome (Leica ultracut UCT, Austria) primary thick sections were prepared, further 70–90 nm ultra-thin sections were prepared (PowerTome PC, RMC Boeckeler, USA) for TEM analysis (Zakaria et al. 2007), whereas 150 nm thick sections were prepared for STEM-EDX (Pagnucco et al. 2023).

### Statistical analysis

Statistical analysis of the data was conducted using independent-sample student t-test to compare the means between two variables (e.g., control vs. Cr(VI) concentration; aerobic vs. anaerobic and between the two different strains). One-way analysis of variance (ANOVA) was applied to compare the means across three or more groups depending on the experimental factors. This was followed by Tukey’s HSD Post Hoc multiple comparison analysis to determine significance differences (*p* < 0.05) among different incubations at different time points using IBM SPSS statistical package (version 21).

## Results

### Growth characteristics of the isolated bacteria

**Fig. 1:**
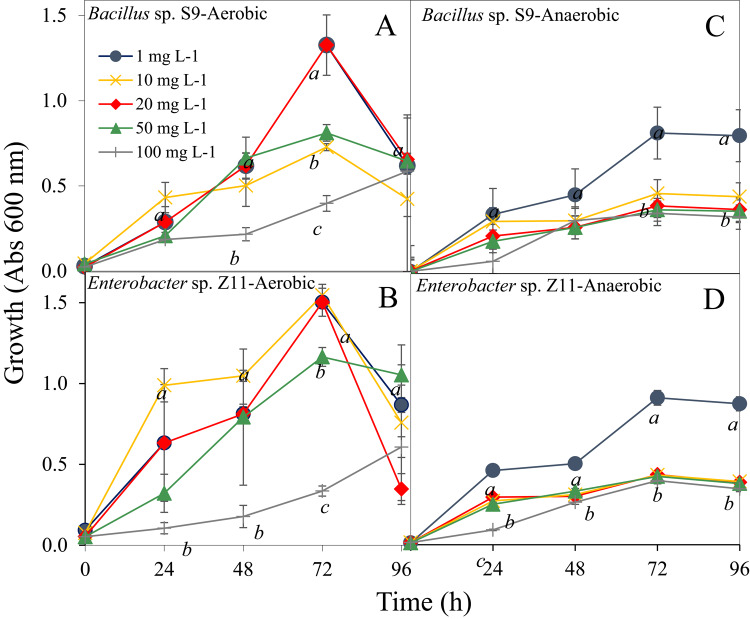
Growth of *Bacillus* sp. S9 and *Enterobacter* sp. Z11 exposed to varying concentrations (1, 10, 20, 50, and 100 mg L^-1^) of Cr(VI) under aerobic (**A**, **B**) and anaerobic (**C**, **D**) conditions over 96 h. Growth was measured as optical density at 600 nm (Abs 600 nm). Symbols represent different concentrations: blue circles (1 mg L^-1^), yellow crosses (10 mg L^-1^), red diamonds (20 mg L^-1^), green triangles (50 mg L^-1^), and gray lines (100 mg L^-1^). Data points show mean values with error bars indicating standard error of the mean (*n* = 3). Different lowercase letters above data points denote statistically significant differences at each time point, as determined by ANOVA followed by Tukey’s HSD test (*p* < 0.05); values sharing the same letter are not significantly different from each other

The two facultative anaerobic bacterial strains, *Bacillus* sp. S9 and *Enterobacter* sp. Z11, grew well under aerobic as well as anaerobic conditions. The optimum growth of both strains, when grown on LB medium, was at pH 9 with 3% salinity at 35 °C (Table S1). Both strains demonstrated the ability to grow and tolerate Cr(VI) at concentrations between 1 and 100 mg L^− 1^ under aerobic as well as anaerobic conditions (Table S1, Fig. 1). As expected, their growth in the presence of oxygen (Fig. 1A, B) was significantly higher than in the absence of oxygen (*p* < 0.01; Fig. 1C, D). Under anaerobic conditions, growth of the strains was significantly highest (OD = 0.84 ± 0.05) at 1 mg L^− 1^ (*p* < 0.001), but decreased with increasing Cr(VI) concentrations (Fig. 1C, D). This was supported by the logistic growth model (Table S2), which revealed that lower Cr(VI) concentrations (1 to 50 mg L^− 1^) supported moderate growth capacities and rates of both strains under aerobic conditions, with the highest concentration (100 mg L^− 1^) induced delayed and abnormal growth patterns, reflecting stress and toxicity effects. Model parameters further demonstrated that Cr(VI) reduced maximum growth rates (µmax) and extended lag phases (λ) under aerobic conditions (Fig. 1A, B), with the most pronounced effects observed at 100 mg L^-1^. These findings confirm metal-induced stress in both strains. In contrast, anaerobic growth (Fig. 1C, D) showed reduced overall growth levels and more pronounced inhibitory effects of Cr(VI), with slower growth rates and longer lag phases across all concentrations. These data collectively indicate that Cr(VI) exerted a dose-dependent inhibitory effect on bacterial growth, with aerobic conditions offering more resilience compared to anaerobic environments.

### Bioreduction of Cr(VI) in the cell-free supernatant

**Fig. 2:**
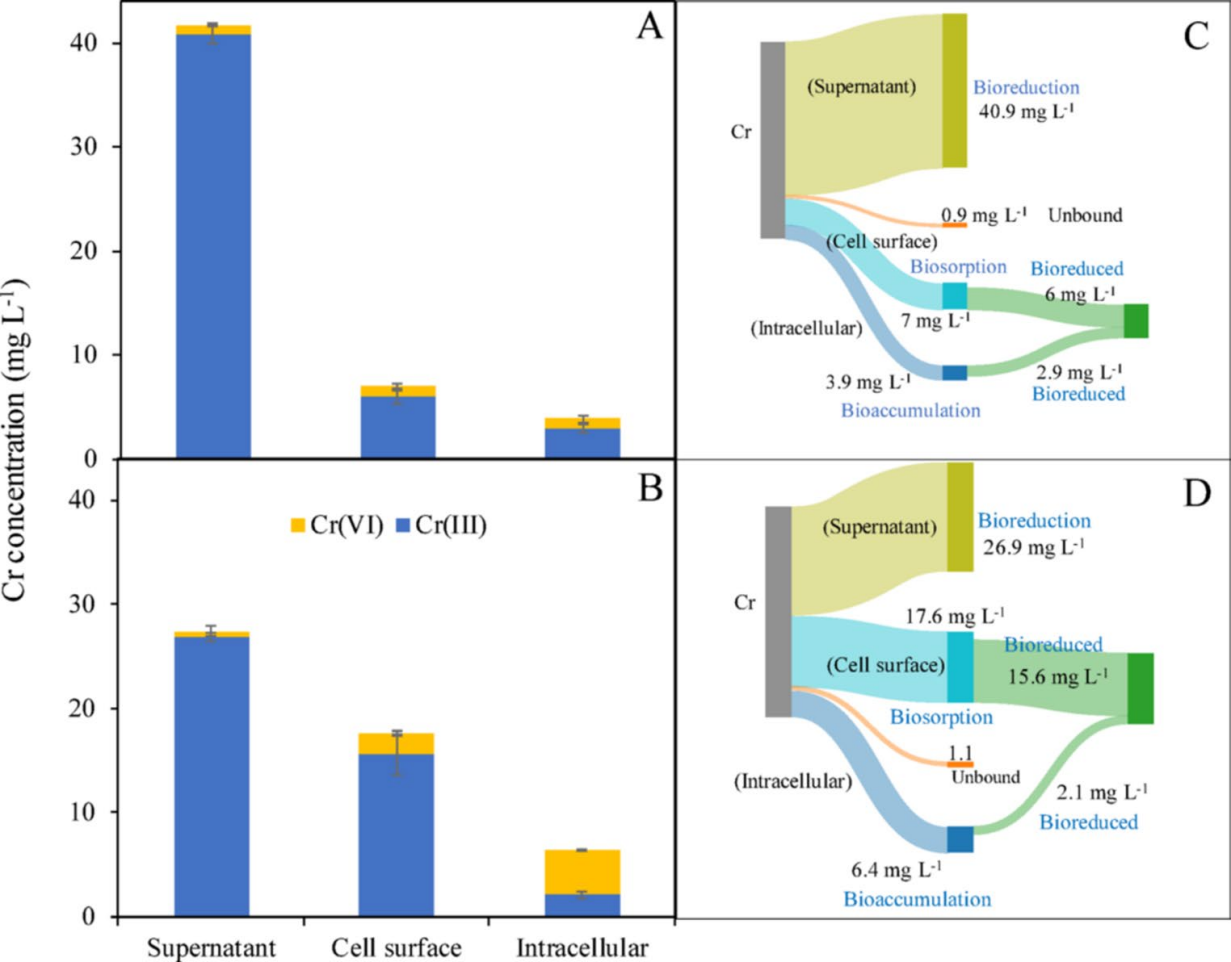
Distribution and transformation of Cr species in *Bacillus* sp. S9, and *Enterobacter* sp. Z11. Panels **A** (*Bacillus* sp. S9) and **B** (*Enterobacter* sp. Z11) show the concentration of Cr(VI) (yellow bars) and Cr(III) (blue bars) in three fractions: supernatant, cell surface, and intracellular compartments after exposure to 50 mg L^− 1^ of Cr(VI) for 7 days under anaerobic conditions. Data represent mean values with standard error of the mean (*n* = 3). Panels **C** (*Bacillus* sp. S9), and **D** (*Enterobacter* sp. Z11) present Sankey diagrams illustrating the fate of added Cr(VI) in the bacterial cultures. The diagrams depict the total amount (in mg L⁻¹) detected in supernatant [bioreduced from Cr(VI) to Cr(III)], cell surface [either biosorbed on the surface or further reduced to Cr(III)], and intracellular [bioaccumulated or further reduced to Cr(III)]

At the beginning of the experiment, 50 mg L^− 1^ Cr(VI) was added, however, the initial measured concentration directly after addition was 52.7 ± 0.05 mg L^− 1^ for *Bacillus* sp. S9 and 52 ± 0.5 mg L^− 1^ for *Enterobacter* sp. Z11 (Table 1), which could be attributed to technical and analytical reasons. The recovered concentrations after 7 days incubations were 51.8 ± 0.6 and 50.9 ± 0.9 mg L^− 1^ for both strains, respectively, which corresponded to ca. 98 ± 0.01% of the initially measured amounts. At the end of the Cr(VI) removal experiment, both Cr(VI) and Cr(III) were detected in the supernatant, on the cell surface, and intracellularly (Table 1; Fig. 2A, B). In the supernatant, bioreduction of Cr(VI) to Cr(III) was significantly higher in *Bacillus* sp. S9 than in *Enterobacter* sp. Z11 (*p* < 0.001, Table 1; Fig. 2A, B). In the case of *Bacillus* sp. S9, only 0.9 ± 0.05 mg L^− 1^ of Cr(VI) was still detectable in the supernatant at the end of the incubation, whereas 40.9 ± 1 mg L^− 1^ of Cr(VI) was reduced to Cr(III) (Fig. 2A, C). This corresponds to the reduction of 77.6 ± 1% of the initial concentration of Cr(VI) at a removal rate of 5.8 ± 0.1 mg L^− 1^ d^− 1^ (Table 1). *Enterobacter* sp. Z11 reduced 26.9 ± 0.4 mg L^− 1^ of Cr(VI) to Cr(III) at a removal rate of 3.8 ± 0.06 mg L^− 1^ d^− 1^ (Table 1; Fig. 2B, D), while 1.1 ± 0.1 mg L^− 1^ remained as Cr(VI) in the supernatant (Table 1).

**Table 1:**
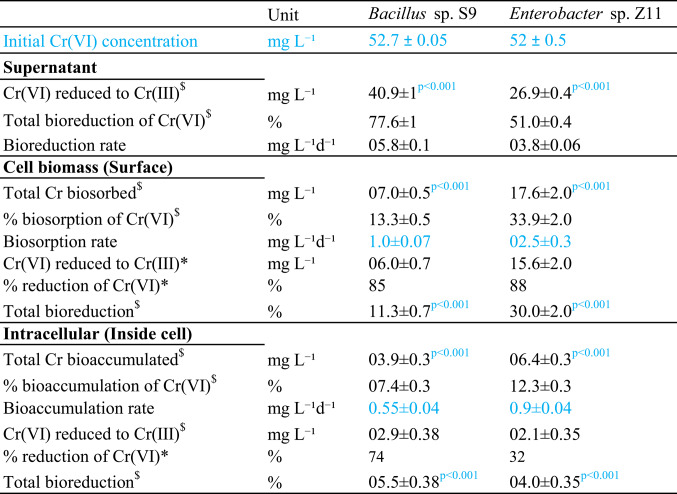
Cr(VI) removal by *Bacillus* sp. S9 and *Enterobacter *sp. Z11 grown anaerobically in the presence of Cr(VI) through reduction to Cr(III), biosorption, and bioaccumulation in the supernatant, on cell surfaces, and intracellularly. *Bacillus* sp. S9 showed higher Cr(VI) reduction in the supernatant, while *Enterobacter* sp. Z11 demonstrated greater biosorption and bioaccumulation. Rates and total bioreduction contributions varied by compartment and strain, with significant differences (*p* < 0.001) noted. All incubations were done in triplicate. Tolerance ± margin of error

### Biosorption and bioreduction of Cr(VI) by cell biomass

**Fig. 3:**
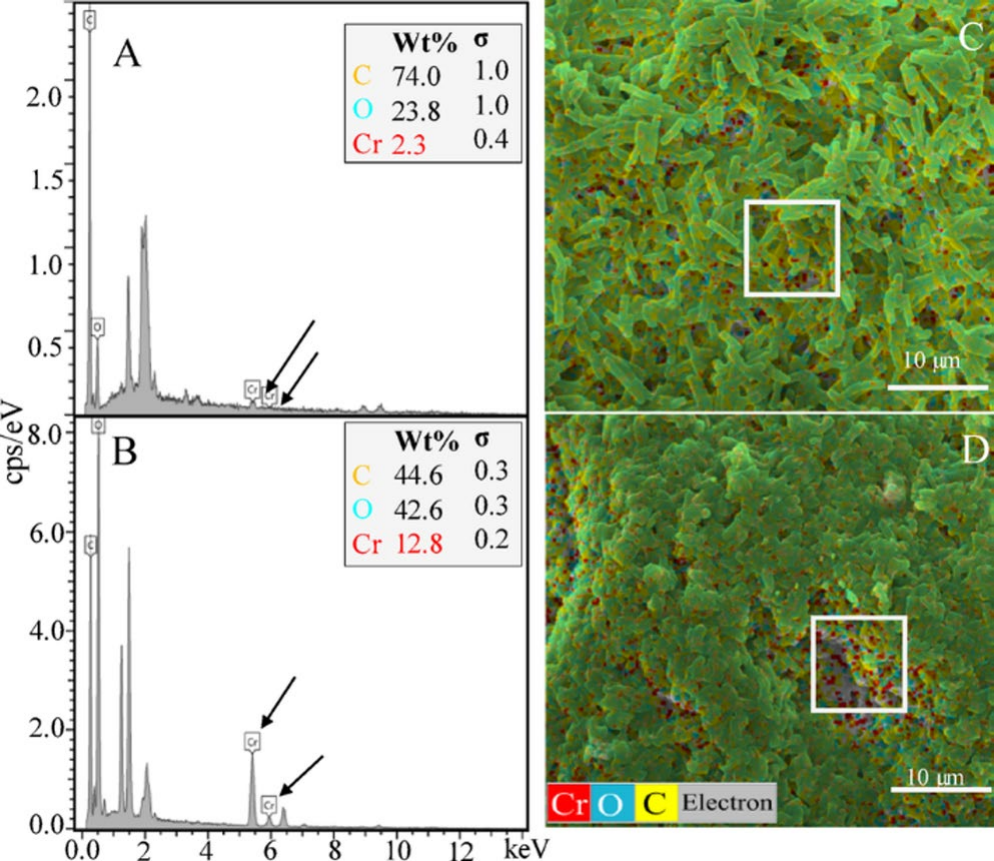
SEM-EDX spectra (A, B) and elemental maps (C, D) of *Bacillus* sp. S9 (**A**, **C**) and *Enterobacter* sp. Z11 (**B**, **D**) after 7 days incubation with 50 mg L⁻¹ Cr(VI). Arrows in **A** and **B** indicate Cr peaks in EDX spectra. Tables show elemental composition (weight% and standard deviation) of regions within the white boxes in **C** and **D**, where Cr (red), O (blue), and C (yellow) are mapped on bacterial surfaces. Scale bar in **C** and **D** is 10 μm. Images represent typical results from at least three experiments; no statistical indicators are shown

**Fig. 4:**
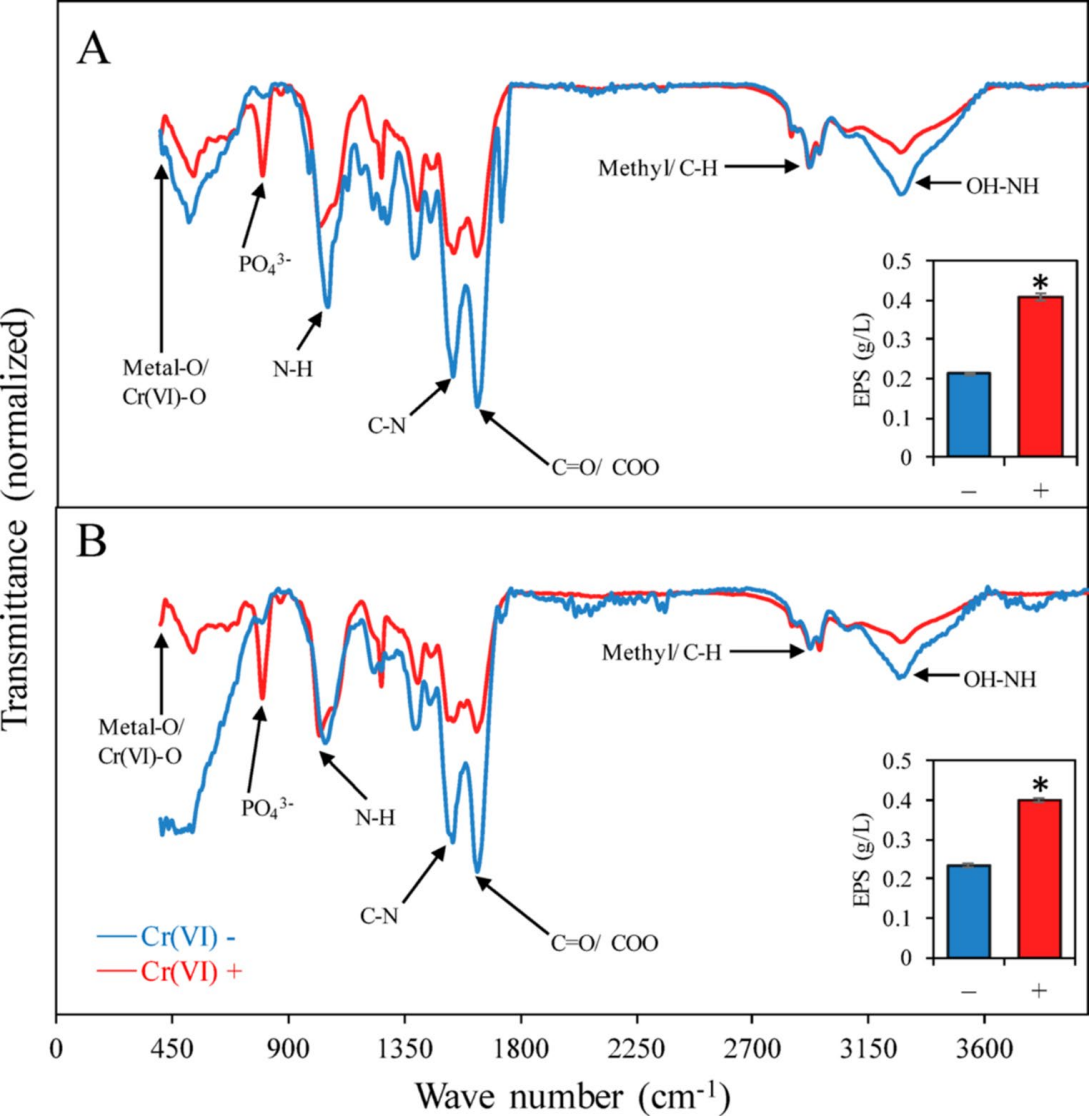
FTIR spectra of biomass harvested from anaerobic cultures of *Bacillus* sp. S9 (**A**) and *Enterobacter* sp. Z11 (**B**) after 7 days of incubation in the absence [blue line, Cr(VI)–] and presence [red line, Cr(VI)+] of 50 mg L^-1 ^Cr(VI). The spectra reveal changes in functional groups associated with cell surface components and EPS upon exposure to Cr(VI). Insets show the quantification of EPS secretion by each strain in the presence and absence of Cr(VI). Bars represent mean values ± standard error (*n* = 3). Asterisks (*) indicate statistically significant differences between treatments (*p* < 0.05, Student’s t-test)

Higher amounts of Cr were detected on the cell surface of *Enterobacter* sp. Z11 (17.6 ± 2 mg L^− 1^) compared to *Bacillus* sp. S9 (7 ± 0.5 mg L^− 1^, *p* < 0.001), accounting for 33.9 ± 2% and 13.3 ± 0.5% of total added Cr, respectively (Table 1; Fig. 2C, D). SEM-EDX analysis confirmed the biosorption of Cr on the outer surface of *Bacillus* sp. S9 as well as *Enterobacter* sp. Z11 (Fig. 3A, B). The SEM-EDX of the control samples exhibited no Cr on the surface of the bacteria (data not shown). Elemental mapping of metals further validated this absorption by confirming the identity of Cr (Fig. 3C, D). FTIR spectroscopy identified the functional groups that could potentially play a role in the biosorption process (Fig. 4). FTIR analysis revealed distinct spectral differences between bacterial cultures incubated with and without Cr(VI) (Fig. A, B). A broad absorption band observed in the 3200–3400 cm⁻¹ region was assigned to overlapping O–H and N–H stretching vibrations, corresponding to contributions from hydroxyl groups (e.g., carbohydrates) and amines/proteins. The absorption attributed to methyl/C–H groups, indicated in the 2800–3000 cm⁻¹ range, originated from C–H stretching vibrations, which is consistent with the methyl (–CH₃) and methylene (–CH₂) groups from lipids and membranes (Fig. A, B). The spectra also displayed features corresponding to C = O and COO stretches (around 1640–1800 cm⁻¹), indicative of carbonyl and carboxylate groups, including amide I bands from proteins. Bands near 1630 cm⁻¹ were assigned to C = O stretching, while those near 1540 cm⁻¹ corresponded to N–H bending with C–N stretching, characteristic of the amide II region of proteins. Peaks identified at lower wave lengths, such as those attributed to C–N, C–O, and PO₄³⁻ stretching, confirmed the presence of protein, nucleic acid, and phospholipid groups. Distinct absorbances near 400–900 cm⁻¹ were associated with Metal–O and Cr(VI)–O vibrations, confirming chromate interactions.

EPS secretion, which facilitates biosorption, exhibited a significant two-fold increase in production upon exposure to 50 mg L^− 1^ of Cr(VI) in both strains under anaerobic conditions (inserts in Fig. 4A, B, *p* < 0.01). In *Bacillus* sp. S9, EPS contained 0.73 ± 0.05 g L^− 1^ proteins and 3.12 ± 0.5 g L^− 1^ carbohydrates, whereas in *Enterobacter* sp. Z11, EPS contained 0.63 ± 0.05 g L^− 1^ proteins and 3.32 ± 0.5 g L^− 1^ carbohydrates.

**Fig. 5:**
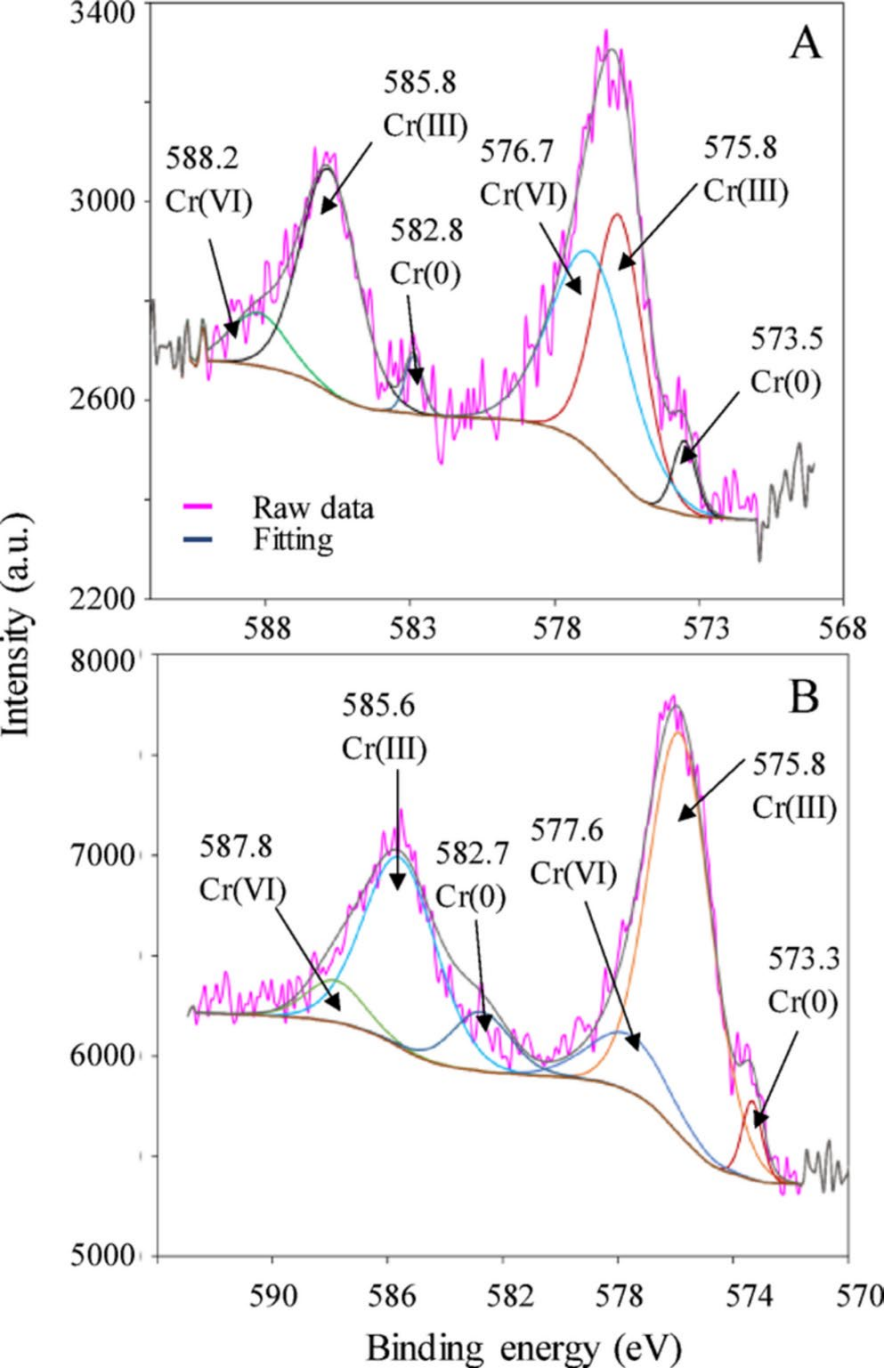
XPS spectra of the Cr 2p region obtained from biomass of *Bacillus* sp. S9 (**A**) and *Enterobacter* sp. Z11 (**B**) after 7 days of incubation with 50 mg L⁻¹ Cr(VI) under anaerobic conditions. The pink lines represent the raw experimental data, while the blue lines indicate the fitted spectra. Deconvoluted peaks correspond to Cr(VI), Cr(III), and elemental Cr(0), with their respective binding energies labeled in electron volts (eV). The spectra confirmed the reduction of Cr(VI) to Cr(III) and further transformation into Cr(0). Peak fitting was performed using Gaussian–Lorentzian functions with a Shirley background subtraction. The results shown are representative of three independent replicates, and peak positions were consistent across replicates (variation < 0.2 eV)

Most of the detected Cr on the cell surface of *Enterobacter* sp. Z11 compared to *Bacillus* sp. S9 was in the form of Cr(III), accounting for 88% (15.6 ± 2 out of 17.6 ± 2 mg L^− 1^) and 85% (6 ± 0.7 out of 7 ± 0.5 mg L^− 1^) of the total detected Cr on the surface, respectively (Table 1; Fig. 2C and D). The bioreduction of Cr(VI) to Cr(III) at the cell surface was further confirmed using XPS analysis on the collected biomass (pellet) at the end of the experiment (Fig. 5A, B). In the case of *Bacillus* sp. S9, the Cr 2P spectrum displayed two prominent peaks around 576 eV and 586 eV for Cr 2p^3/2^ and Cr 2p^1/2^, respectively (Chen et al. 2018). The deconvolution of the peak at 576 eV (Cr 2p^3/2^) and 586 eV revealed 3 peaks each (Fig. 5A). The peaks at 575.8 (Cr 2P^3/2^) and 585.8 eV (Cr 2P^1/2^) were attributed to Cr(III) while the peaks at 576.7 (Cr 2P^3/2^) and 588.2 eV (Cr 2P^1/2^) referred to Cr(VI). Elemental Cr(0) was identified at 573.5 eV (Cr 2P^3/2^) and at 582.8 eV (Cr 2p^1/2^) (Huang et al. 2024; Salvi et al. 1995) (Fig. 5A). For Cr 2P^3/2^, Cr(0) accounted for 5.6% of total Cr while Cr(III) and Cr(VI) accounted for 42.8% and 51.6% of total Cr, respectively. For Cr 2P^1/2^, Cr(0), Cr(III) and Cr(VI) accounted for 6.0, 75.9 and 18.1%, respectively. In case of *Enterobacter* sp. Z11, Cr 2P showed two main peaks around 576 eV and 587 eV for Cr 2p^3/2^ and Cr 2p^1/2^, respectively. The deconvolution of the peak at 576 eV and 587 eV showed 3 peaks each (Fig. 5B). The peaks at 573.3 eV (Cr 2p^3/2^) and at 582.7 (Cr 2P^1/2^) were related to Cr(0) (Huang et al. 2024; Salvi et al. 1995). The peaks at 575.8 (Cr 2P^3/2^) and 585.6 eV (Cr 2P^1/2^) referred to Cr(III), while the peaks at 577.6 (Cr 2P^3/2^) and 587.8 eV (Cr 2P^1/2^) referred to Cr(VI) (Fig. 5B). For Cr 2P^3/2^, Cr(0) accounted for 5.03% of total Cr while Cr(III) and Cr(VI) accounted for 80.5 and 14.4% of total Cr, respectively. For Cr 2P^1/2^, Cr(0), Cr(III) and Cr(VI) accounted for 18.3, 68.9 and 12.8%, respectively (Fig. 5B).

Bioreduction of Cr(VI) to Cr(III) was also confirmed using the chromate reductase enzyme assay performed on CFE from collected biomass. The total protein content was 0.167 g L^− 1^ for *Bacillus* sp. S9 and 0.078 g L^− 1^ for *Enterobacter* sp. Z11. *Bacillus* sp. S9 and *Enterobacter* sp. Z11 required 2 mL of the CFE to achieve 100% removal for 1 mg L^− 1^ Cr(VI). The calculated specific activities for *Bacillus* sp. S9 and *Enterobacter* sp. Z11 were 1.92 U/mg and 4.11 U/mg protein, respectively.

### Intracellular bioaccumulation

**Fig. 6:**
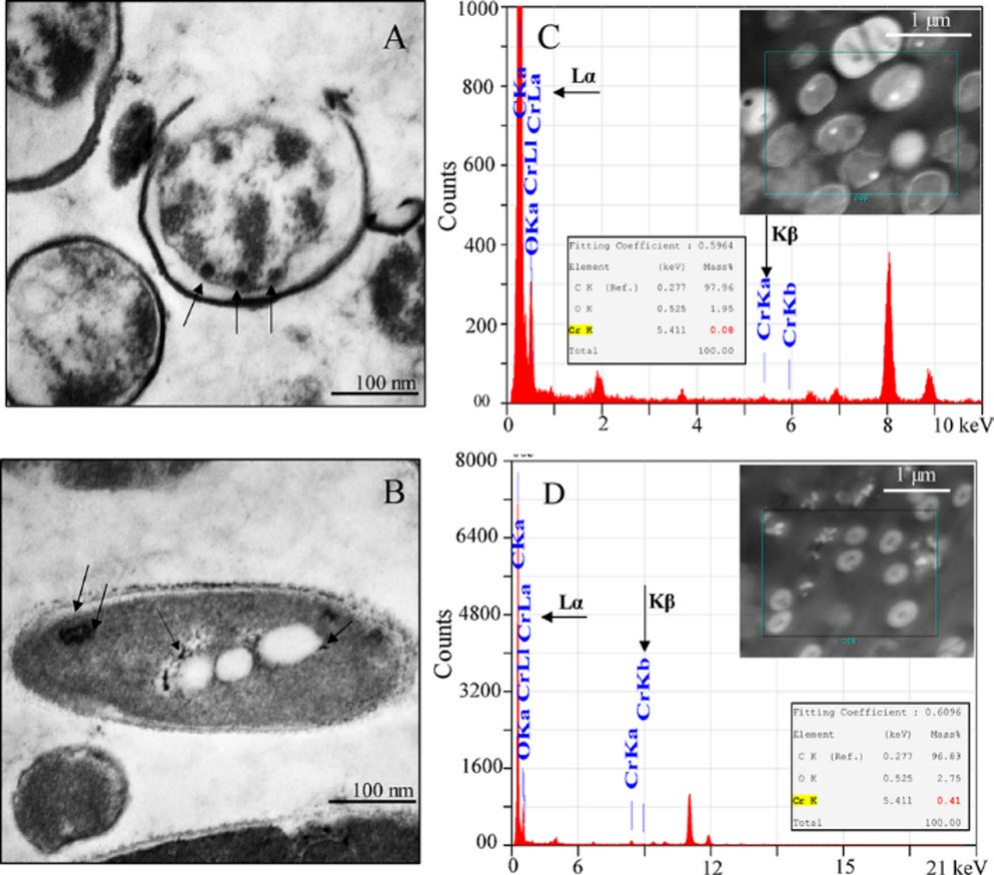
TEM images of *Bacillus* sp. S9 (**A**) and *Enterobacter* sp. Z11 (**B**) after 7 days of incubation with 50 mg L⁻¹ Cr(VI) under anaerobic conditions. Black arrows indicate electron-dense intracellular deposits corresponding to Cr accumulation. Panels (**C**) and (**D**) show STEM-EDX of thin cell sections from *Bacillus* sp. S9 and *Enterobacter* sp. Z11, respectively. The insert images display the analyzed regions (highlighted by boxes). The EDX spectra confirm the presence of Cr, with characteristic Cr Lα and Cr Kβ peaks detected alongside carbon (C) and oxygen (O) signals from the cellular matrix. Quantitative elemental composition (expressed as weight%) is shown in the embedded tables, indicating significant Cr enrichment within the cells. Scale bars: 100 nm (**A**, **B**) and 1 μm (**C**,** D**)

After cell digestion using 1% HNO_3_, Cr was detected inside the cells of both strains. A total amount of 3.9 ± 0.3 (i.e. 7.4 ± 0.3% of total amount), and 6.4 ± 0.3 mg L^− 1^ (i.e. 12.3 ± 0.3% of total amount) Cr bioaccumulated in the cells of *Bacillus* sp. S9 and *Enterobacter* sp. Z11, respectively (Table 1; Fig. 2C and D). This difference in the amount of intracellularly bioaccumulated Cr in both strains was statistically validated (*p* < 0.001). While Cr(III) constituted most of the detected Cr inside the cells of *Bacillus* sp. S9 (74%), it made up only 32% in *Enterobacter* sp. Z11 (Table 1). The intracellular bioaccumulation of Cr by both strains was validated using TEM analysis (Fig. 6A, B). Upon exposure to 50 mg L^− 1^ Cr(VI) under anaerobic conditions, both strains exhibited circular electron-dense black spots in the cytoplasm (Fig. 6A, B). These dark intracellular inclusions were consistently observed across representative cells in multiple fields examined, and have been suspected to represent Cr precipitates. STEM-EDX analysis confirmed Cr presence associated with these inclusions, supporting the bioaccumulation of Cr inside the cells of both strains (Fig. 6C, D). The mass % of Cr in the STEM-EDX images was ca. 0.41% in *Enterobacter* sp. Z11 (insert in Fig. 6D), and ca. 0.08% in *Bacillus* sp. S9 (insert in Fig. 6C) indicating higher bioaccumulation in the former organism. Staining of lipids in bacterial cells using Sudan III showed their accumulation in *Enterobacter* sp. Z11 under Cr(VI) stress. The bacterial cells (blue) and lipid spots (orange) were observed inside the cells, as highlighted by black arrows in Supplementary Figure S2.

## Discussion

Our findings demonstrated that the two facultative anaerobic bacteria, *Bacillus* sp. S9 and *Enterobacter* sp. Z11 were capable of removing Cr(VI) under anaerobic conditions through bioreduction, biosorption, and bioaccumulation. The isolates belonged to the phyla Firmicutes (*Bacillus* sp. S9) and Proteobacteria (*Enterobacter* sp. Z11), which have been predominantly detected in the original mat (Abed et al. 2020; Khan et al. 2025a). Their growth characteristics are commensurate with the environmental conditions at the sampling site (Abed et al. 2020). Although our strains were isolated from a photosynthetic microbial mat near a chromite mining site, they were phylogenetically related to bacteria previously isolated from tannery and industrial effluents (Sharma et al. 2008; Vidali 2001; Zha et al. 2024). Microbes that survive in mine drainage are expected to tolerate high metal concentrations (Banerjee and Dey 2024). Our strains exhibited detectable growth with up to 100 mg L^−1^ Cr(VI), suggesting that they could tolerate even higher concentrations. While contamination levels in natural environments are often lower and subject to seasonal and spatial fluctuations, similar or even higher Cr(VI) concentrations have been reported in heavily polluted industrial effluents and landfill leachates (Banchor et al. 2020; Abed et al. 2020; Kholisa and Chirwa 2021), supporting the relevance of our study’s concentration choice for extreme contamination scenarios. For example, an industrial landfill leachate in Bhilai, India contained Cr(VI) concentrations as high as 1050 mg L^−1^, with surface water showing up to about 22 mg L^−1^ in post-monsoon seasons (Banchor et al. 2020). Similarly, microbial mats from Cr mining quarry sumps in Oman showed natural Cr content around 1911 mg kg^−1^ (Abed et al. 2020). Different bacteria employ different mechanisms to tolerate or detoxify Cr(VI) including the production of metal-binding proteins, efflux pumps, or specific enzymes (Deo et al. 2024; Narayani and Shetty 2013). However, growth decline at high concentrations could be due to Cr(VI) toxic effects. High levels of Cr(VI) can disrupt cellular processes and induce oxidative stress, likely due to the generation of reactive oxygen species (Deo et al. 2024; Narayani and Shetty 2013).

### Bioreduction of Cr(VI) to Cr(III)

ICP-OES analysis of both forms of Cr in the supernatant, cell surface, and inside the cells as well as XPS data and chromate reductase assay provided multiple lines of evidence of biologically-mediated enzymatic mechanism for reduction of Cr(VI) to Cr(III) by *Bacillus* sp. S9 and *Enterobacter* sp. Z11. Dissimilatory bioreduction of Cr(VI) to Cr(III) was described as the most common mechanism adopted by bacteria for detoxification of Cr(VI) (Deo et al. 2024; Narayani and Shetty 2013; Thatoi et al. 2014). Reduction of Cr(VI) to Cr(III) can occur intracellularly after Cr(VI) uptake through sulfate transporters, or extracellularly via secreted metabolites or surface-associated enzymes (Bandara et al. 2020; Xu et al. 2011; Li et al. 2023; Rahman and Thomas 2021). While our isolates reduced Cr(VI) in the supernatant at the rates of 5.8 ± 0.1 mg L^− 1^ d^− 1^ and 3.8 ± 0.06 mg L^− 1^ d^− 1^, for *Bacillus* sp. S9 and *Enterobacter* sp. Z11, respectively, the removal rates for other strains were different depending on the initial Cr(VI) concentrations, incubation conditions, and the type of bacterial strain. For instance, members of the *Enterobacteriaceae* family were able to remove 99% of 13 mg L^− 1^ Cr(VI) at a removal rate of 0.56 mg L^− 1^ d^− 1^ (Martins et al. 2010). The anaerobic facultative bacterium *Exiguobacterium* sp. PY14 exhibited a maximum Cr(VI) tolerance of 400 mg L^− 1^ Cr(VI), and removed 12.5% at a removal rate of 8.33 mg L^− 1^ d^− 1^ (Huang et al. 2023). The strain *Sporosarcina saromenis* W5 was reported to remove 200 mg L^− 1^ Cr(VI) at the rate of 0.25 mg L^− 1^ d^− 1^ (Huang et al. 2021).

Chromate reductase enzyme assay performed on CFE from collected biomass of our strains confirmed the involvement of the enzyme in reducing Cr(VI) to Cr(III). In fact, previous research has demonstrated the induction and expression of chromate reductase gene in both aerobic as well as facultative anaerobic bacteria when grown in the presence of Cr(VI) (Narayani and Shetty 2013; Thatoi et al. 2014). Several strains of the bacterial genera *Pseudomonas*, *Bacillus*, and *Arthrobacter*, were shown to reduce Cr(VI) using enzymes encoded by plasmid-borne or chromosomal genes (Deo et al. 2024; Narayani and Shetty 2013; Thatoi et al. 2014). Chromate reductase not only promoted Cr(VI) removal and detoxification but also improved cell survival by minimizing oxidative stress (Deo et al. 2024; Narayani and Shetty 2013; Thatoi et al. 2014). While the specific activities of the CFE (enzyme) were 1.92 U/mg protein for *Bacillus* sp. S9 and 4.11 U/mg protein for *Enterobacter* sp. Z11, previous studies have reported a range of enzyme activities in different bacterial strains using CFEs (Ontañon et al. 2018; Ouled-Haddar et al. 2018; Camargo et al. 2003). For instance, *Bacillus* sp. SFC 500-1E showed a specific enzyme activity of 3.7 U/mg protein (Ontañon et al. 2018). In another study, the enzyme activity of different strains of *Lactobacilli* was compared, with the highest reported value of 1.52 U/mg protein for *L. casei* (Mishra et al. 2012). *Bacillus megaterium* A3-1 showed an enzyme activity of 0.003 U/mg protein (Ouled-Haddar et al. 2018), whereas five soil-isolated *Bacillus* species reported specific activities in CFEs from 0.34 to 0.48 U/mg protein (Camargo et al. 2003). These comparisons indicate that the chromate reductase activities observed in *Bacillus* sp. S9 and *Enterobacter* sp. Z11 were notably higher than many previously reported values, suggesting a potentially greater efficiency of these strains in Cr(VI) reduction. In some bacteria, like *Arthrobacter* sp. and *Bacillus amyloliquefaciens*, chromate reductase activity has been detected extracellularly (Narayani and Shetty 2013; Thatoi et al. 2014), which is consistent with the detection of chromate reductase activity in CFEs of *Bacillus* sp. S9 and *Enterobacter* sp. Z11 cultures. These findings are also in line with the detection of Cr(III) in cell-free culture supernatants (Aslam et al. 2020; Camargo et al. 2003).

XPS analysis confirmed the reduction of Cr(VI) to Cr(III), but suggested the presence of a minor fraction of elemental chromium Cr(0). While the reduction of Cr(VI) to Cr(0) is thermodynamically feasible under strongly reducing conditions, this process is rare and not well documented in biological systems, due to the very low redox potential required and the absence of known enzymes catalyzing this reaction. A few studies have reported the formation of Cr(0) in the presence of strong electron donors like H_2_ or formate under strict anaerobic conditions (Li et al. 2023). It was proposed that this reduction was predominantly abiotic, potentially mediated by products of microbial metabolites or biogenic compounds such as cytochromes or H_2_S. For instance, a study performed using the facultative anaerobe *Bacillus subtilis* SL-44 reported on the formation of Cr(0) when incubated with Cr(VI) and humic acid under anaerobic conditions (Li et al. 2023). However, we acknowledge that our interpretation of Cr(0) presence from XPS data remains speculative, and future work including experiments with cell-free supernatants, filtrates, and heat-killed controls is needed to rigorously test the role of biogenic mediators in Cr(0) formation.

### Biosorption of Cr(VI)

Binding of Cr(VI) on the cell surface of *Bacillus* sp. S9 and *Enterobacter* sp. Z11 was confirmed through SEM-EDX analysis, and the involved functional groups have been identified by FTIR. Biosorption is likely the initial mechanism employed by microbes to remove Cr(VI), because cell membranes are the first point of contact with the metal. Hence, surface biosorption plays a vital role in Cr(VI) removal (Deo et al. 2024; Narayani and Shetty 2013; Pagnucco et al. 2023), and has been described in many Cr(VI)-removing aerobic and anaerobic bacteria (Aishwarya et al. 2024; Huang et al. 2021, 2023; Li et al. 2023; Pagnucco et al. 2023). A comparison between the amounts of Cr(VI) adsorbed on the surfaces of different bacteria when incubated at 50 mg L^− 1^ Cr(VI) under anaerobic conditions highlights the superior biosorption capabilities of our strains. For instance, while a total amount of 7 ± 0.5 mg L^− 1^ and 17.6 ± 2 mg L^− 1^ was adsorbed on the surface of *Bacillus* sp. S9 and *Enterobacter* sp. Z11, respectively, the strains *Sporosarcina saromensis* and *Exiguobacterium* sp. adsorbed only 3.139 mg L^− 1^ and 1.5 mg L^− 1^, respectively when incubated at the same (pH = 9, Temp = 35 °C and initial concentration = 50 mg L^− 1^, anaerobic) conditions (Huang et al. 2021, 2023). However, it should be kept in mind that the biosorption capacity of different microbes is influenced by their metabolic activities, surface proteins, as well as EPS production (Bales et al. 2013; Gupta and Diwan 2017). It is well established that the cell membrane of Gram-positive bacteria (e.g. *Bacillus* sp. S9) are significantly different than that of Gram-negative bacteria (e.g. *Enterobacter* sp. Z11). In fact, Gram-positive bacteria are generally expected to exhibit greater metal-binding capacity, owing to the thick and porous peptidoglycan layer in their cell wall, which offers a large surface area and abundant binding sites. Additionally, the high abundance of the negatively charged teichoic acids and the presence of various functional groups on the peptidoglycan matrix (e.g., carboxyl, hydroxyl, and amine groups) further enhance their metal-binding capacity (Gupta and Diwan 2017). Nevertheless, the amount of Cr adsorbed on the cell surface of *Bacillus* sp. S9 was lower than that of *Enterobacter* sp. Z11. One possible explanation is that *Bacillus* sp. S9 may have initially adsorbed more Cr(VI) on its surface, which was then partially reduced to Cr(III) over the course of the experiment. However, this remains a hypothesis as our study only provides end-point measurements, and future work incorporating kinetic data will be required to confirm the adsorption and reduction dynamics. Nevertheless, *Bacillus* sp. S9 appears to be more effective in overall Cr(VI) removal compared to *Enterobacter* sp. Z11 under the tested conditions.

Our FTIR data showed consistent spectral alterations compared to previous studies at bands corresponding to OH-NH, methyl-C-H, C = O/COO, C-N, N-H, PO_4_^3−^, metal–O, and Cr(VI)–O, confirming the direct involvement of these groups in Cr(VI) binding on the surface (Deo et al. 2024; Yang et al. 2024; Dey 2022; Shukla et al. 2012). Cr(VI) bioreduction to Cr(III) apparently involved surface oxidation, as evidenced by the appearance of the O–H peak at 3274 cm⁻¹, the carboxyl (-COOH) peak at 1724 cm⁻¹, and the increased intensity of C–O bands between 1000 and 1400 cm⁻¹ (El-Shafey et al. 2016; Al-Shamakhi et al. 2022; Zlotnikov et al. 2023). Previous studies demonstrated the involvement of similar functional groups in both Gram-positive and Gram-negative bacteria despite the prominent variations in their cell wall composition (Banerjee and Dey 2024; Deo et al. 2024; Huang et al. 2021; Narayani and Shetty 2013). These groups originate from proteins, polysaccharides, lipids, and teichoic acids in Gram-positive bacteria or lipopolysaccharides and phospholipids in Gram-negative bacteria. However, the abundance, accessibility, and contribution to Cr(VI) binding can differ due to variations in cell wall architecture. The detection of metal-O and Cr(VI)-O groups on the surface is noteworthy, as these spectral signatures are associated with studies involving Cr removal by dead biomass or EPS, and are rarely reported in living cells (Gupta and Diwan 2017; Shukla et al. 2012). It is plausible that these functional groups originate from EPS produced in response to Cr(VI) exposure. This assumption is consistent with previous findings indicating EPS involvement in metal binding (Abed et al. 2020; Khan et al. 2025a; Pagliaccia et al. 2022; Pagnucco et al. 2023). The two fold increase in EPS when both strains were exposed to Cr(VI) suggests that this is a common adaptive mechanism to Cr(VI) stress (Gupta and Diwan 2017; Harish et al. 2012).

### Bioaccumulation of Cr(VI)

Cr(VI) bioaccumulation inside the cells in the form of intracellular inclusion bodies or by specific metal-binding proteins is consistent with observations in previously investigated strains (Middleton et al. 2003; Pagnucco et al. 2023). Previous reports demonstrated the ability of different bacteria to bioaccumulate Cr(VI) into their cells (Aslam et al. 2020; Essahale et al. 2012; Ouled-Haddar et al. 2018; Zakaria et al. 2007). For instance, *Acinetobacter haemolyticus* and *Shewanella oneidensis* MR-1 have been shown to form intracellular and extracellular electron-dense Cr precipitates upon incubation in Cr(VI) (Middleton et al. 2003; Pagnucco et al. 2023; Zakaria et al. 2007). While *Bacillus* sp. S9 and *Enterobacter* sp. Z11 bioaccumulated 3.9 ± 0.3 mg L^− 1^ and 6.4 ± 0.3 mg L^− 1^ Cr(VI), respectively, earlier studies reported significantly higher accumulation levels (Aslam et al. 2020; Ouled-Haddar et al. 2018). For example, *Bacillus megaterium* A3-1 accumulated 15.7 mg Cr g^− 1^ when exposed to 30 mg L^− 1^ Cr(VI), accounting for approximately 50% of the initial concentration (Ouled-Haddar et al. 2018). On the other hand, *Klebsiella pneumoniae* accumulated 83.5% of Cr(VI) when incubated at the concentration of 0.2 mg L^− 1^ (Aslam et al. 2020). The lower bioaccumulation of Cr(VI) in our strains was attributed to the fact that a significant portion of Cr(VI) was adsorbed on the cell surface and reduced to Cr(III). The detection of Cr(III) inside the cells was indeed validated by ICP-OES analysis after cell digestion. STEM-EDX analysis revealed not only the uptake of Cr inside the cell, but also the formation of distinct inclusions, which were later confirmed by Sudan-III test (Supplementary Fig. S2) to constitute lipids. Exposure to heavy metals is known to increase lipid accumulation in bacterial cells (Lara et al. 2021; Middleton et al. 2003; Zakaria et al. 2007). Co-localization mapping with elemental overlays is still required to confirm the nature and identity of the Cr inclusions, and to obtain quantitative data on the extent of Cr bioaccumulation in bacterial cells.

## Conclusion

The facultative anaerobic strains *Bacillus* sp. S9 and *Enterobacter* sp. Z11 isolated from a phototrophic microbial mat from a mining site exhibited strong Cr(VI) removal capacity under anaerobic conditions by employing at least three distinct mechanisms: biosorption, bioaccumulation, and bioreduction (Fig. 3S). Overall, *Bacillus* sp. S9 had a higher removal capacity than *Enterobacter* sp. Z11. Upon exposure to Cr(VI), the metal was immediately adsorbed to the cell surface, followed by induction of chromate reductase enzyme that reduced Cr(VI) to Cr(III), while a fraction of the metal was accumulated inside the cell. Additionally, EPS secreted by these strains facilitated effective trapping of Cr(VI) at the cell surface, thereby increasing the overall efficiency of Cr(VI) removal. The ability of facultative anaerobic bacteria to efficiently remove Cr(VI) underscores their adaptability and strong potential as candidates for bioremediation in both aerobic and anaerobic metal-contaminated environments. For field application, Cr(VI) removal by microorganisms could be achieved in scalable bioreactors or by in situ applications, such as injection wells creating microbial filters for groundwater treatment. Nonetheless, performance will be influenced by many factors including competition with native microbial communities, fluctuating nutrient and electron donor levels, and the influence of physicochemical conditions such as pH and redox state. These factors must be considered when laboratory-based results are translated to field-scale bioremediation application, and additional research involving pilot-scale and in situ trials are necessary to optimize and validate this biotechnological approach.

## Supplementary Information

Below is the link to the electronic supplementary material.

Supplementary Material 1

## Data Availability

All relevant data are within the paper.
