# Supplementary material for: Removal of hexavalent chromium by facultative anaerobic strains Bacillus sp. S9 and Enterobacter sp. Z11 from a mining site microbial mat
Source: 3 Biotech. 2026 May 15;16(6):199. doi: 10.1007/s13205-026-04841-9 (PMC13179404; doi:10.1007/s13205-026-04841-9)
Supplement: Supplementary file 1 — Supplementary Material 1 [file 13205_2026_4841_MOESM1_ESM.pptx]

## Slide 1
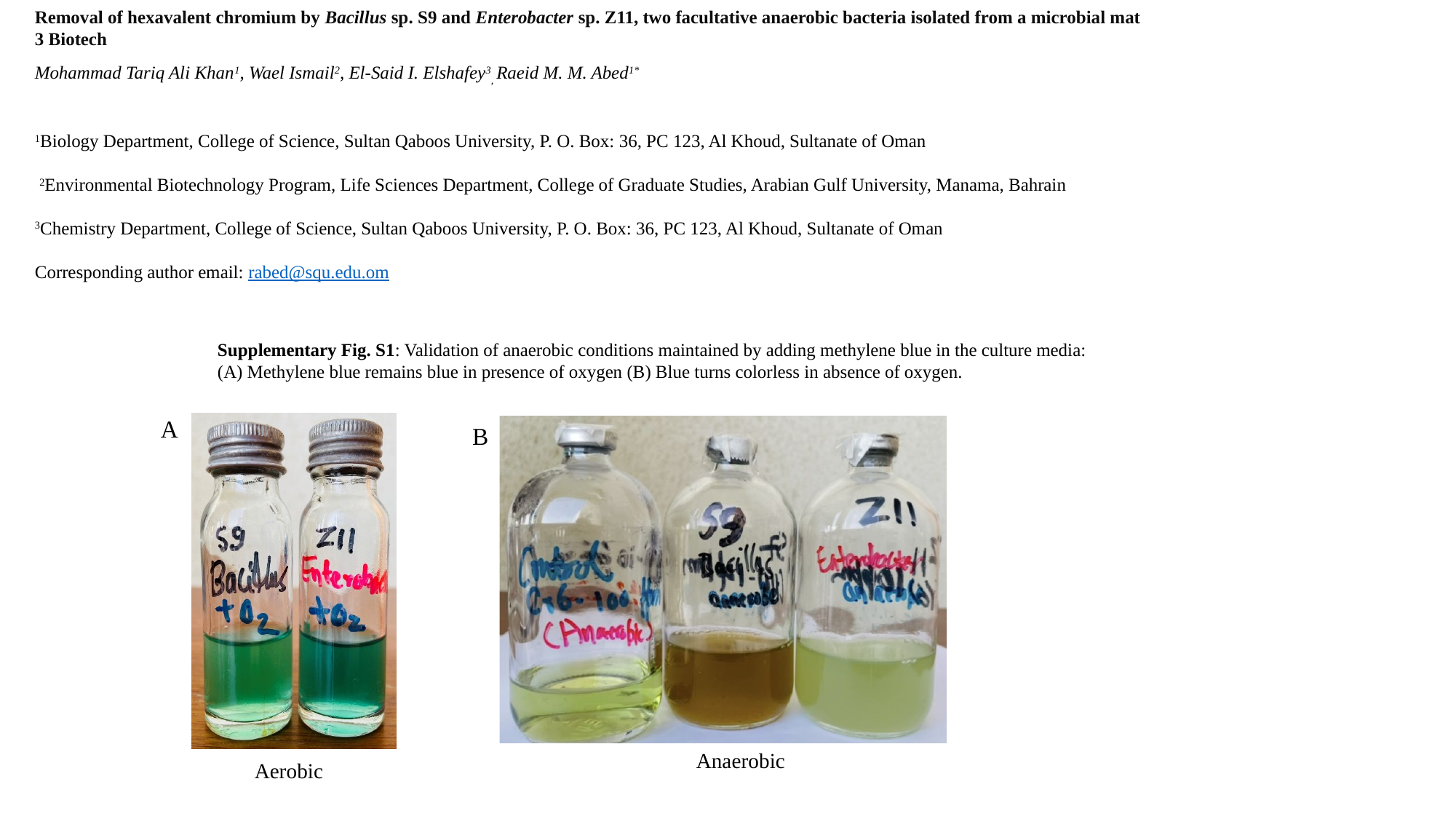

Removal of hexavalent chromium by Bacillus sp. S9 and Enterobacter sp. Z11, two facultative anaerobic bacteria isolated from a microbial mat
3 Biotech
Mohammad Tariq Ali Khan1, Wael Ismail2, El-Said I. Elshafey3, Raeid M. M. Abed1*
1Biology Department, College of Science, Sultan Qaboos University, P. O. Box: 36, PC 123, Al Khoud, Sultanate of Oman
 2Environmental Biotechnology Program, Life Sciences Department, College of Graduate Studies, Arabian Gulf University, Manama, Bahrain
3Chemistry Department, College of Science, Sultan Qaboos University, P. O. Box: 36, PC 123, Al Khoud, Sultanate of Oman
Corresponding author email: rabed@squ.edu.om
Supplementary Fig. S1: Validation of anaerobic conditions maintained by adding methylene blue in the culture media:
(A) Methylene blue remains blue in presence of oxygen (B) Blue turns colorless in absence of oxygen.
A
B
Anaerobic
Aerobic

## Slide 2
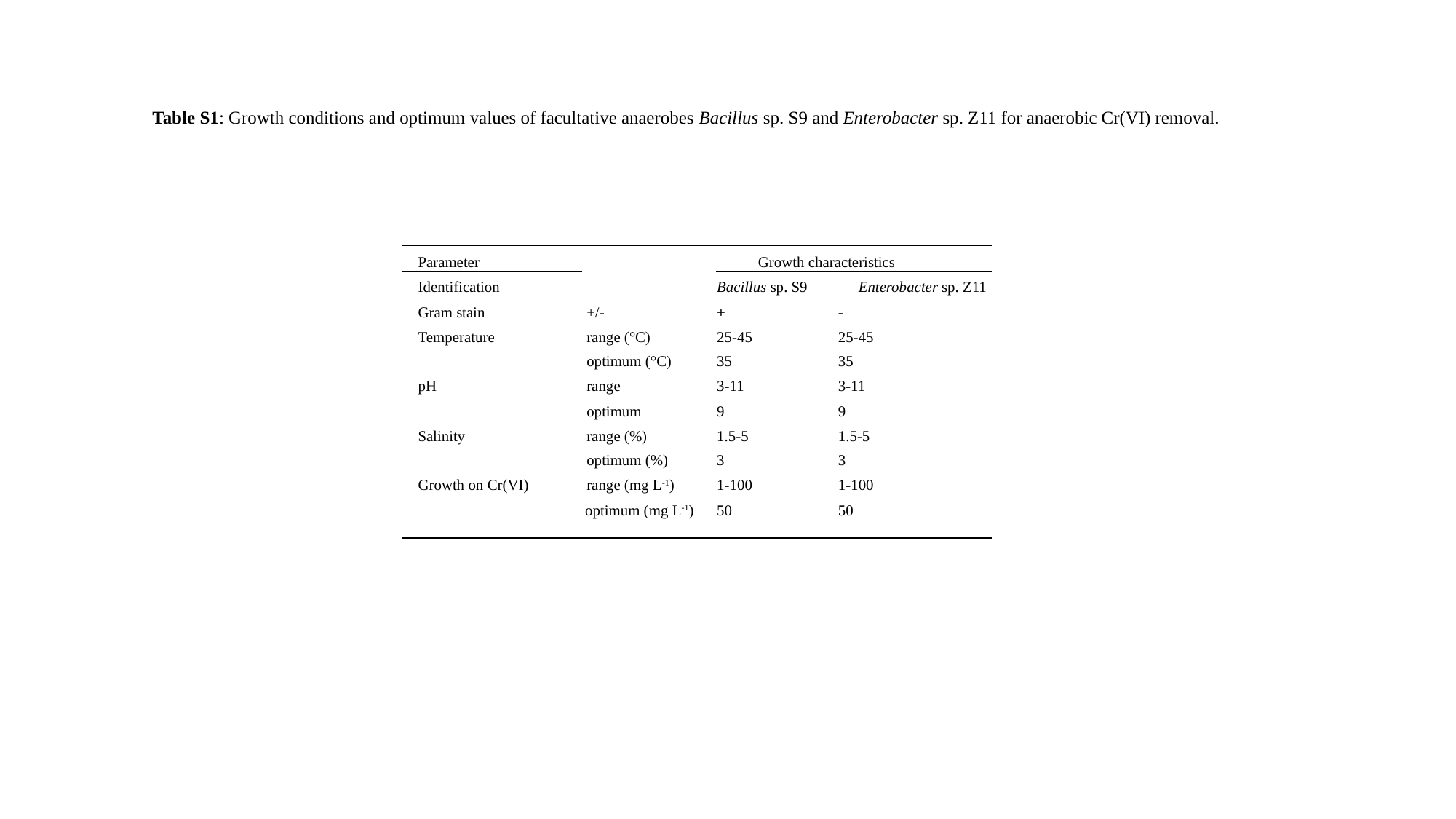

Table S1: Growth conditions and optimum values of facultative anaerobes Bacillus sp. S9 and Enterobacter sp. Z11 for anaerobic Cr(VI) removal.
| Parameter | | Growth characteristics | | |
| --- | --- | --- | --- | --- |
| Identification | | Bacillus sp. S9 | Enterobacter sp. Z11 | |
| Gram stain | +/- | + | - | |
| Temperature | range (°C) | 25-45 | 25-45 | |
| | optimum (°C) | 35 | 35 | |
| pH | range | 3-11 | 3-11 | |
| | optimum | 9 | 9 | |
| Salinity | range (%) | 1.5-5 | 1.5-5 | |
| | optimum (%) | 3 | 3 | |
| Growth on Cr(VI) | range (mg L-1) | 1-100 | 1-100 | |
| | optimum (mg L-1) | 50 | 50 | |
| | | | | |

## Slide 3
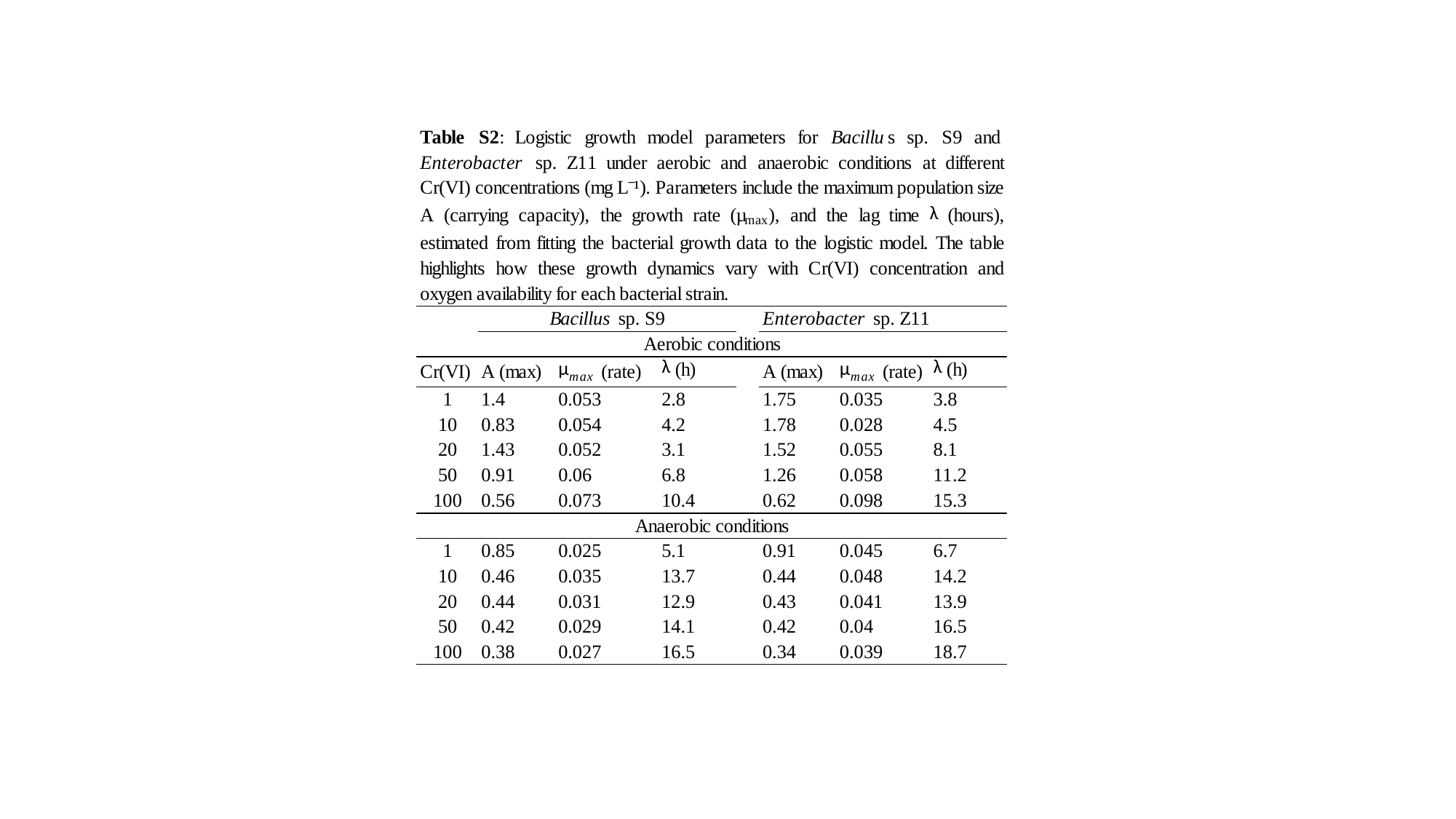

## Slide 4
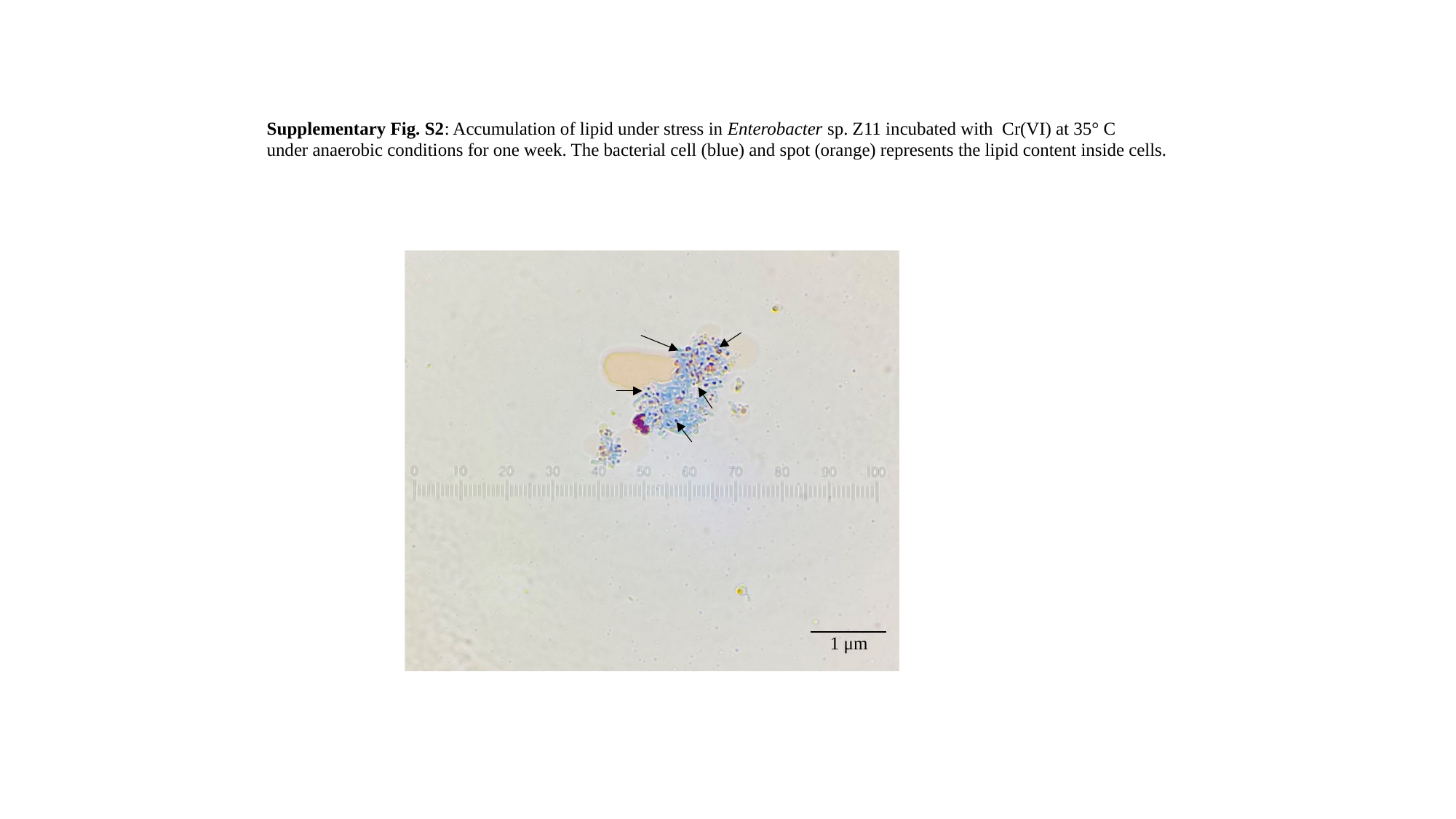

Supplementary Fig. S2: Accumulation of lipid under stress in Enterobacter sp. Z11 incubated with Cr(VI) at 35° C
under anaerobic conditions for one week. The bacterial cell (blue) and spot (orange) represents the lipid content inside cells.
1 μm

## Slide 5
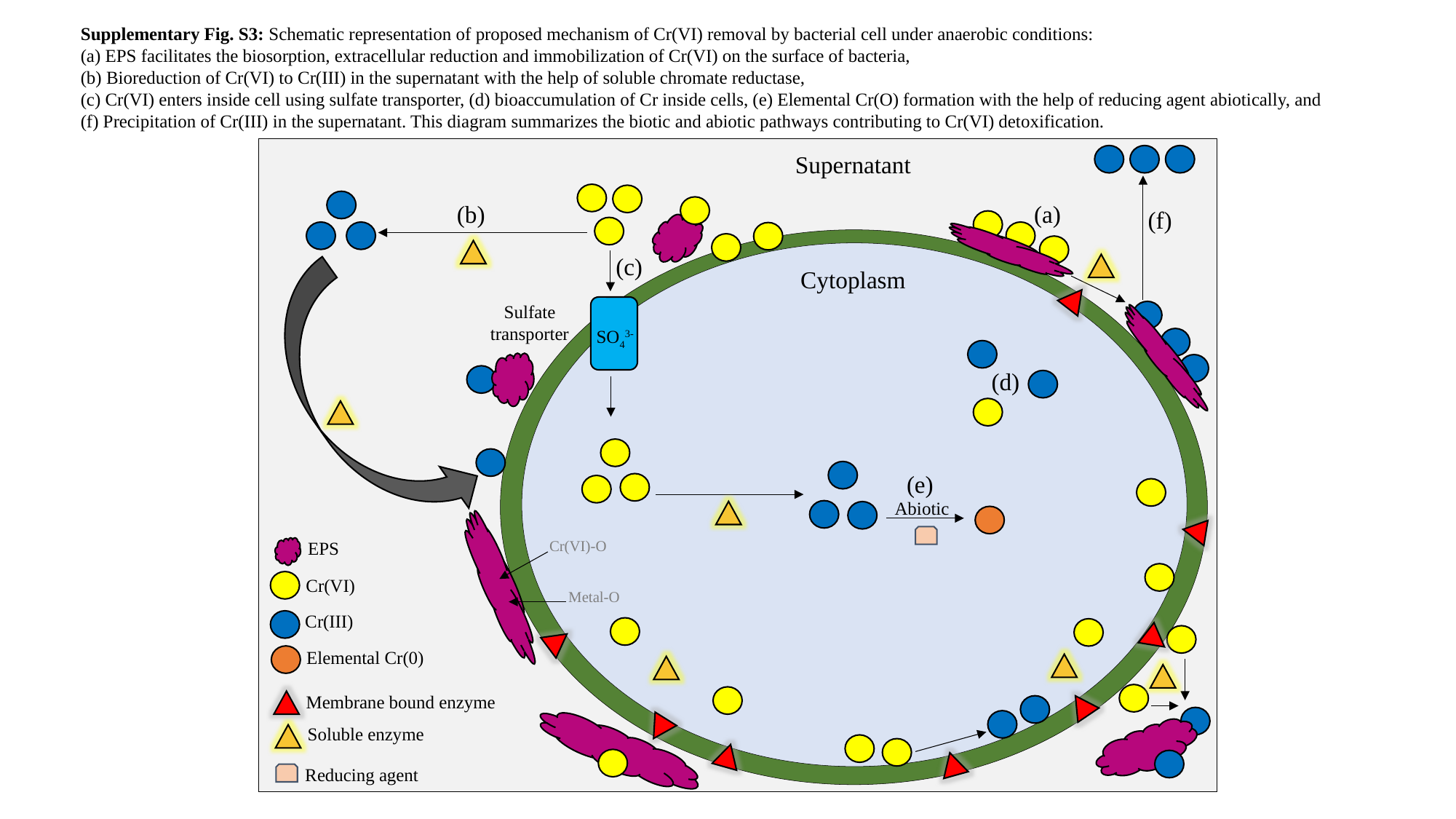

Supplementary Fig. S3: Schematic representation of proposed mechanism of Cr(VI) removal by bacterial cell under anaerobic conditions:
(a) EPS facilitates the biosorption, extracellular reduction and immobilization of Cr(VI) on the surface of bacteria,
(b) Bioreduction of Cr(VI) to Cr(III) in the supernatant with the help of soluble chromate reductase,
(c) Cr(VI) enters inside cell using sulfate transporter, (d) bioaccumulation of Cr inside cells, (e) Elemental Cr(O) formation with the help of reducing agent abiotically, and
(f) Precipitation of Cr(III) in the supernatant. This diagram summarizes the biotic and abiotic pathways contributing to Cr(VI) detoxification.
Supernatant
(a)
(b)
(f)
(c)
Cytoplasm
Sulfate
transporter
SO43-
(d)
(e)
Abiotic
Cr(VI)
Cr(III)
Elemental Cr(0)
Membrane bound enzyme
Soluble enzyme
Cr(VI)-O
EPS
Metal-O
Reducing agent
